# Comparative ^1^H NMR Metabolomic Urinalysis of People Diagnosed with Balkan Endemic Nephropathy, and Healthy Subjects, in Romania and Bulgaria: A Pilot Study

**DOI:** 10.3390/toxins3070815

**Published:** 2011-07-04

**Authors:** Peter Mantle, Mirela Modalca, Andrew Nicholls, Calin Tatu, Diana Tatu, Draga Toncheva

**Affiliations:** 1 Centre for Environmental Policy, Imperial College London, London SW7 2AZ, UK; 2 Dialysis Center (renamed Medical Service), Drobeta-Turnu Severin RO-220012, Romania; Email: mirela_modilca@yahoo.com; 3 Investigative Preclinical Toxicology, GlaxoSmithKline R&D, Park Road, Ware, Herts SG12 0DP, UK; Email: andrew.w.nicholls@gsk.com; 4 University of Medicine and Pharmacy, Timisoara RO-300708, Romania; Email: geomed88@googlemail.com (C.T.); diana_szilagyi@yahoo.com (D.T.); 5 Department of Medical Genetics, Medical University, Sofia 1431, Bulgaria; Email: dragatoncheva@yahoo.com

**Keywords:** Balkan nephropathy, metabolomics, urinalysis, haemodialysis, ochratoxin A, aristolochic acid

## Abstract

^1^H NMR spectroscopy of urine has been applied to exploring metabolomic differences between people diagnosed with Balkan endemic nephropathy (BEN), and treated by haemodialysis, and those without overt renal disease in Romania and Bulgaria. Convenience sampling was made from patients receiving haemodialysis in hospital and healthy controls in their village. Principal component analysis clustered healthy controls from both countries together. Bulgarian BEN patients clustered separately from controls, though in the same space. However, Romanian BEN patients not only also clustered away from controls but also clustered separately from the BEN patients in Bulgaria. Notably, the urinary metabolomic data of two people sampled as Romanian controls clustered within the Romanian BEN group. One of these had been suspected of incipient symptoms of BEN at the time of selection as a ‘healthy’ control. This implies, at first sight, that metabolomic analysis can be predictive of impending morbidity before conventional criteria can diagnose BEN. Separate clustering of BEN patients from Romania and Bulgaria could indicate difference in aetiology of this particular silent renal atrophy in different geographic foci across the Balkans.

## 1. Introduction

The Balkan endemic nephropathy was first recognised in the mid 1950s as a slow, progressive and silent morbidity in certain rural communities in Bulgaria, the former Yugoslavia and Romania [1,2,3,4,5]. It is characterised by gradual deterioration of urinary excretion, and is confirmed post-mortem by the profound atrophy of both kidneys. An increased incidence of tumours of the urinary tract is associated with the nephropathy, but this does not include renal cell carcinoma. Many studies have since sought to discover the cause of this uremic disease, with many theories passing in and out of favour, but there seems still no satisfactory identification of the causal factor(s). Marked histopathological changes have already occurred by the time the morbidity is diagnosed and therefore the course of early stages remains unclear. Particularly there are no biomarkers specific to the very early stage of disease. 

Development of metabolomics, from the early discovery of the analytical power of ^1^H NMR spectroscopy of small volumes of human and experimental animal blood and urine [6,7], offers potential for recognition of disorder by combinations of biomarkers. Combining the advantage of several years of involvement with the disease with opportunity to apply for the first time the new field of metabolomics, an informal international collaboration was established in 2003 to explore urine of Balkan nephropathy patients and of apparently health control subjects in regions of Bulgaria and Romania where the nephropathy has been and remains hyperendemic. 

## 2. Materials and Methods

### 2.1. Romania: Balkan Nephropathy

Urine of 21 BEN patients was collected at the Dialysis Center in Drobeta Turnu Severin during attendance for routine haemodialysis in May 2003 (15 subjects) or as the second urination of the day after overnight stay in hospital (6 subjects). The samples were representative of a typical routine day at that clinic. All urine samples were collected into 15 mL calibrated Falcon tubes containing 1 mL 1% sodium azide solution. 

Four additional samples were obtained from the same hospital in 2004; two samples came from females which provided samples 12 and 18 in the 2003 group, and one of these was currently receiving peritoneal dialysis (Supplementary Table 4). The other two were additions to the study which was therefore conducted on 25 urine samples (M:F ratio 10:15) from 24 separate BEN subjects. One sample came from a patient who had been admitted to hospital during the night following a home visit from several of us. None of the persons was suspected of having a urinary tract tumour.

### 2.2. Romania: Control

Control samples (18) were obtained a year later (21–22 April 2004), partly (13 subjects) in the historically (since 1957)-hyperendemic nephropathy village of Erghevita where residents had become accustomed over many years to medico-scientific visits concerning BEN. Subjects were selected as convenient, arising voluntarily from casual informal interaction with residents during the visit. Five samples were also taken from a family adjacent to a BEN-family in a nearby village. Age and general features of health were noted during private conversation with one of us (D.T., who is a medical doctor). Thus we ensured that only persons without any history of nephropathy, and not currently taking prescribed medication, were asked to provide a sample. All conversants were co-operative, since this did not require any invasive procedure. Sample composition was therefore conditioned mainly according to fluid intake during the first half of the day; samples from Erghevita are mostly listed (Supplementary Table 3) in chronological order of collection. For example, sample 6 was collected at 10 a.m., samples 7 and 8 around 11 a.m., samples 9 and 10 at mid-day (subjects had recently consumed beer), samples 12–16 during early afternoon, samples 11,17,18 later in afternoon. Samples (~10 mL) were immediately mixed with 1 % sodium azide solution (1 mL) as above.

Whereas, for obtaining the nephropathy samples the subjects were self-selected according to visiting the hospital for haemodialysis, subjects for the control samples were also self-selected by availability in routine village life, including typical consumption of beer by some residents on a hot day. Notably, however, after informal “consultation” with one person in Erghevita, D.T. spontaneously expressed the clinical opinion that that person could already unwittingly be showing clinical signs of BEN. None of the persons was suspected of having a urinary tract tumour.

### 2.3. Bulgaria: Balkan Nephropathy and Controls

Groups of samples from BEN patients (case 19, Supplementary Table 2), receiving haemodialysis, and apparently healthy controls (case 21, Supplementary Table 1) were obtained from females in endemic villages in the Vratza region from the second urination of the day after overnight fast. Freshly collected urine (5–10 mL) was added to 1 mL 1% sodium azide solution as preservative in a graduated plastic tube. None of the persons was suspected of having a urinary tract tumour.

### 2.4. Romania: Patients with Urological Disease not Associated with BEN

Since there was no overt evidence of urinary tract tumours developing in any of the subjects sampled in the categories above, urine samples were collected according to the protocol above at the County Hospital, Timisoara in October 2003 from seven local residents with urological disease requiring surgery. Timisoara is ~200 Km north-west of the nephropathy area and the subjects had no diagnosis involving BEN. The samples were collected during days prior to the surgical procedure and served as disease-controls in relation to the samples collected from BEN patients in 2.1 above. Samples were from six males and one female (age range 58–75 years) with tumours in prostate, kidney, bladder, upper urinary tract and periuretral tissues.

### 2.5. Urinalysis

Urinalysis for creatinine, calcium, urate, protein, sodium, potassium, phosphate and urea was made by standard automated methodology [8] in the Chemical Pathology Department at St Mary’s Hospital, London. Data adjustment was made according to the urine dilution effected by the azide preservative. It was not possible to know whether any creatinine concentration values varied atypically due to excessive or limited recent consumption of liquid, but concentration values for all other analysed parameters in each sample were adjusted relative to 1 mmol creatinine, to allow comparability across samples.

### 2.6. ^1^H NMR Spectroscopic Analysis of Urine

Each sample (400µL) was mixed with 200 µL of phosphate buffer (0.2 M NaH_2_PO_4_: 0.2 M Na_2_HPO_4_ (19:81), pH 7.4). Aliquots of the resulting mixture (500 mL) were placed in 5 mm NMR tubes to which 50 mL of a solution of TSP in D_2_O was added (final concentration, 1 mM). The D_2_O plus TSP addition provided both a chemical shift reference (δ 0.0) and a field frequency lock signal. One-dimensional (1D) ^1^H NMR spectra were acquired at 699.93 MHz on a Bruker Avance 700 spectrometer using a standard pre-saturation pulse sequence for water suppression with solvent irradiation in the relaxation delay (3 s) and the mixing time (100 ms). NMR spectra were acquired using 128 scans into 64 k points with a spectral width of 14097 Hz, an acquisition time of 2.32 s, and a total pulse recycle delay of 5.42 s. The FIDs were multiplied by an exponential function corresponding to a 0.3 Hz line broadening prior to Fourier transformation. All data were phased and baseline corrected manually in TopSpin (version 2.5, Bruker GmbH, Germany). 

### 2.7. Multivariate Statistical Analysis of 1H NMR Spectral Data

All NMR spectra were processed for multivariate statistical analysis using the AMIX software (version 3.9.7, Bruker GmbH, Germany). The spectra were normalised to the total integral (excluding water, urea and TSP) and data reduced to a series of 0.02 ppm integrated regions for analysis. The reduced dataset was pre-processed (pareto scaling) and analysed via Principal Component Analysis (PCA) and Partial Least Squares–Discriminant Analysis (PLS-DA) using the SIMCA−P+ software package (version 11.5, Umetrics, Umea, Sweden). Comparisons were made between the data from the two groups of control subjects and the two groups of subjects diagnosed with Balkan Endemic Nephropathy (BEN). Those regions of the NMR spectra causing clustering in the data were identified from the coefficients. The metabolites were assigned based on comparison to authentic standards, existing data and on-line resources. 

## 3. Results

**Figure 1:**
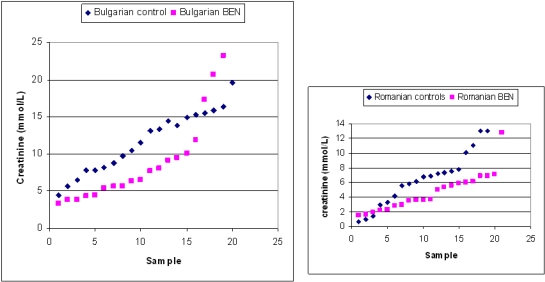
Comparisons of urinary creatinine concentrations in sample groups sourced in Bulgaria and Romania showing dominance of lower values for people diagnosed with BEN. Illustration of Bulgarian data omitted one exceptionally high value (42.5 mmol/L) for a control subject, which otherwise distorts the graphical format. The three lowest values for the Romanian controls were from subjects who had recently been drinking.

Analysis of several standard urinary parameters indicative of renal function, particularly when expressed relative to a unitary value of creatinine concentration, defined the status of each subject concerning the urine sample studied also by ^1^H NMR spectroscopy. Table 1 summarises the analytical findings; the detail can be seen in Tables 1–5 in Supplementary data. Considerable variation in creatinine concentration in controls from both Balkan countries and elsewhere is normal for human populations. An important factor is the extent of liquid intake in the preceding few hours. Therefore the rather similar distribution ranges, expressed both in the present apparently healthy controls (Supplementary Tables 1 and 3), and in the BEN subjects just before receiving one of the thrice-weekly sessions of haemodialysis (Supplementary Tables 2 and 4), confirms the value of the former as controls and indicates the general success of haemodialysis in sustaining adequate renal function in spite of a diagnosis of BEN. However, the range of population values for creatinine concentration, as directly measured for control and BEN groups, distinguished control and BEN groups in each country by the dominance of diluted urine in most of the BEN subjects (Figure 1). The three lowest values for the Romanian controls were from subjects who had recently been drinking. Four additional BEN samples collected in 2004 (in the range 2–6 mmol/L) conformed to the pattern for 2003. 

**Table 1:** Urinalysis data summarised from detail shown in Tables 1–5 (Supplementary data). Mean values (±SD) for Bulgarian and Romanian control and BEN groups, and a group of non-BEN subjects with urinary tract tumours. Units: creatinine (mmol/L), and other parameters (mmol/mmol creatinine).

		Creatinine	Calcium	Urate	Protein	Sodium	Potassium	Phosphate	Urea
Bulgaria	Control	13.1(7.9)	0.14(0.1)	0.25(0.09)	13.1(7.9)	18.9(8.3)	4.9(3.5)	1.4(0.8)	23.4(8.7)
	BEN	8.8(5.8)	0.24(0.18)	0.2(0.18)	8.8(5.8)	30.0(15.7)	6.2(2.0)	2.6(1.4)	40.3(11.1)
Romania	Control	6.4(3.8)	0.26(0.24)	0.29(0.14)	6.4(3.8)	28.1(13.2)	5.8(2.6)	4.3(4.5)	32.0(12.7)
	BEN	4.4(2.5)	0.14(0.1)	0.2(0.08)	4.4(2.5)	34.6(21.2)	3.8(1.8)	1.8(1.4)	21.2(6.7)
	Non-BEN UTT	11.3(4.2)	0.37(0.37)	0.23(0.1)	11.3(4.2)	19.2(8.4)	2.9(0.6)	2.3(0.9)	46.1(15.6)

Data from all NMR spectra were assessed visually to confirm data quantity following NMR processing, and then explored using multivariate statistical analysis. The initial stage of data analysis included an assessment of those samples found to be strong outliers from the majority of the urinary data. One set of outliers was found to be due to the presence of very high concentrations of ethanol in the samples. This was presumed to be due to alcohol consumption by the subject since no other sources were identified in the collection or analysis stages. Due to the potential for other physiological effects from such consumption, the subject was excluded from the analysis. Similarly, those subjects that showed abnormally high levels of glucose in their urine, without the presence of other metabolites to imply a nephrotoxic event, were considered to be undiagnosed or undeclared diabetes sufferers and were also excluded from the analyses. Further to the exclusion of subjects, signals were observed in the NMR data to imply the presence of paracetamol (acetaminophen). Such background and undeclared use of non-steroidal anti-inflammatory drugs is commonly found in clinical metabolomic assessments. The level of paracetamol signals observed did not imply the sustained use of the drug and, rather than exclude the subjects, those regions of the NMR spectra assigned to the paracetamol signals were excluded from analysis. Furthermore, the regions accounting for the creatinine signals were also excluded since, even with pre-processing, these signals dominated the analysis, thereby obscuring other metabolic alterations. 

An initial PCA assessment of the data was made comparing the samples from the control and BEN subjects from both Romania and Bulgaria along with samples from Romanians with other kidney related disease. The scores from the data showed that the majority of the samples from Romanian BEN patients separated from the samples from all other groups. However, this separation dominated the analysis and all other groups mapped in the same scores space. PCA examination of the data without the Romanian BEN subjects resulted in all groups mapping together in scores space. From examination of the loadings it was apparent that a high degree of variation existed across the samples, which was not representative of any group structure. Some baseline distortion was noted along with some samples showing a dominance of the scores space. Rather than exclude these moderate outliers, it was considered more informative to apply a correlation based analysis.

**Figure 2:**
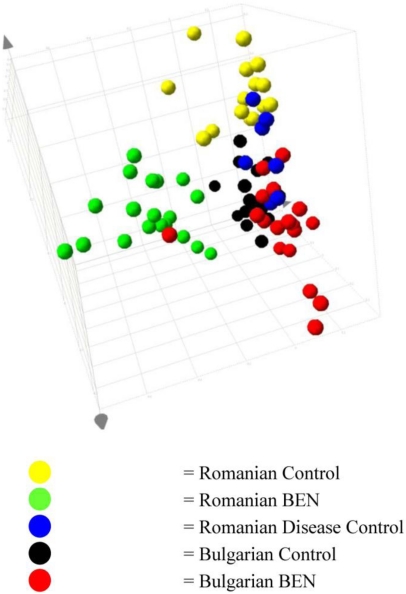
PLS-DA scores plot of the urinary data from Romanian and Bulgarian BEN patients, familial/regional control subjects and subjects with other forms of kidney disease.

PLS-DA analysis of the data from all groups resulted in observation similar to the PCA, with the Romanian BEN subjects separating from the other groups (Figure 2).

When viewed as the plot of the first and second components, one of the samples from the Bulgarian BEN group was also observed to map with the Romanian BEN samples. Three of the Romanian Control samples also showed a trend towards this space. This would infer that the three control Romanians would be showing some metabolic characteristics of BEN, yet had not, at the time of sampling, been clinically diagnosed. The variation in the BEN group was sufficiently great that it dominated the first component. From the coefficients calculated from each variable, those regions of the NMR spectrum of greatest influence to this separation of the Romanian BEN group, and the endogenous metabolites they represented, were identified. 

The separation of the samples from the Romanian BEN group was caused by a urinary increase in glucose, lactate, trimethylamine N-oxide (TMAO) and acetate. The region δ 0.75–1.05 was observed to be increased in the samples from the BEN patients but, due to the extensive overlap of resonances, precise assignment could not be achieved. Typical urine data would contain signals from amino acids, small organic acids and, potentially, protein in this region of the NMR spectrum. Increase to any of these groups would be expected to occur in cases of kidney damage. These changes reflected the general pathophysiological alterations associated with nephrotoxicity or kidney disease. Further to these general alterations, selected metabolites were noted that may be more implicated either as cause or effect of the BEN observed. These included an increase in myo-inositol (δ 4.06, 3.63, 3.55, 3.26) and choline (δ 4.06, 3.55, 3.21). A number of other resonances were noted to be elevated in the samples from the Romanian BEN subjects, but there was insufficient information for assignment. The metabolites, recognised from NMR signals, in these comparisons are listed in Table 2, Table 3, Table 4, Table 5. In comparison, a reduction in the concentration of a number of urinary components was observed, including creatine, phosphocreatine, pyruvate, dimethylglycine, citrate, glycine, succinate, formate and trigonelline.

**Table 2:** Mean fold change and *p* values for metabolites identified to change in urine samples from Romanian BEN subjects compared to samples from Romanian Control subjects.

Metabolite	Mean Fold	*p*	Variation
Protein, amino, organic acids	1.80	4.47 × 10^−6^	elevated
Lactate	1.47	8.30 × 10^−2^	elevated
Unknown triplet	1.57	6.61 × 10^−4^	elevated
Glucose	1.83	1.56 × 10^−4^	elevated
Myo-inositol	1.76	1.27 × 10^−4^	elevated
3-hydroxyisovalerate	1.39	8.88 × 10^−4^	reduced
Pyruvate	1.51	1.67 × 10^−5^	reduced
Citrate	1.93	4.38 × 10^−4^	reduced
Dimethylamine	1.44	7.05 × 10^−5^	reduced
Trimethylamine	1.77	9.40 × 10^−7^	reduced
Dimethylglycine	1.71	7.59 × 10^−5^	reduced
Taurine	1.46	1.31 × 10^−3^	reduced
Glycine	1.28	2.29 × 10^−2^	reduced
Creatine	1.78	1.70 × 10^−6^	reduced
Phosphocreatine	1.49	3.34 × 10^−3^	reduced
Formate	2.47	1.05 × 10^−2^	reduced
Trigonelline	1.99	2.49 × 10^−4^	reduced

**Table 3:** Mean fold change and *p* values for metabolites identified to change in urine samples from Bulgarian BEN subjects compared to samples from Bulgarian Control subjects.

Metabolite	Mean Fold	*p*	Variation
Lactate	1.43	5.93 × 10^−3^	elevated
Citrulline	1.49	7.36 × 10^−3^	elevated
Choline	1.45	2.85 × 10^−2^	elevated
TMAO	2.29	2.89 × 10^−4^	elevated
Citrate	0.67	1.66 × 10^−1^	reduced
Succinate	0.88	8.88 × 10^−2^	reduced
TMA	1.00	3.38 × 10^−1^	reduced
Glycerophosphocholine	0.99	4.92 × 10^−1^	reduced

**Table 4:** Mean fold change and *p* values for metabolites identified to change in urine samples from Romanian Control subjects compared to samples from Bulgarian Control subjects.

Metabolite	Mean Fold	*p*	Variation
Citrate	1.22	1.90 × 10^−1^	elevated
TMA	1.00	2.20 × 10^−1^	elevated
Glycerophosphocholine	0.985472668	0.454055919	elevated
Phenylacetic acid	1.41	1.96 × 10^−2^	elevated
Lactate	1.42	4.92 × 10^−2^	Reduced
Dimethylglycine	1.692875166	0.006970254	Reduced
Choline	1.537199456	0.005899252	Reduced
TMAO	1.588133198	0.009447084	Reduced
Glycine	1.259082095	0.061166452	Reduced
Phosphocreatine	1.559161391	0.003522414	Reduced
Trigonelline	2.193825386	0.001786463	Reduced
Hippurate	1.53	8.45 × 10^−2^	Reduced

**Table 5:** Mean fold change and *p* values for metabolites identified to change in urine samples from Bulgarian BEN subjects compared to samples from Romanian BEN subjects.

Metabolite	Mean Fold	*p*	Variation
Lactate	1.52	7.27 × 10^−3^	Elevated
Acetate	1.36	1.36 × 10^−1^	Elevated
Glucose	1.70	1.49 × 10^−4^	Elevated
Myo-inositol	1.23	4.80 × 10^−3^	Elevated
TMAO	1.28	1.68 × 10^−1^	Elevated
Protein, amino acids and organic acids	1.86	1.50 × 10^−6^	elevated
Citrate	1.80	5.81 × 10^−3^	Reduced
Pyruvate	1.61	2.47 × 10^−7^	Reduced
Taurine	1.85	1.44 × 10^−4^	Reduced
Glycine	1.45	1.92 × 10^−8^	Reduced
Phosphocreatine	1.22	8.26 × 10^−3^	Reduced

Although the separation of the Romanian BEN sufferers represented the greatest variation in the data, sufficient variability existed in the data for the samples from the control and Bulgarian BEN patients to merit examination of the sources of this difference. Figure 4 shows the plot of the second and third components, with the data for the samples from the Romanian BEN patients hidden for clarity. Sufficient variation existed between the groups to give rise to the mapping of the groups in three clearly identifiable regions of the plot. The data indicated that, based on this small set of data, urinary variation enabled identification of the country of origin of the subject providing the sample.

**Figure 3:**
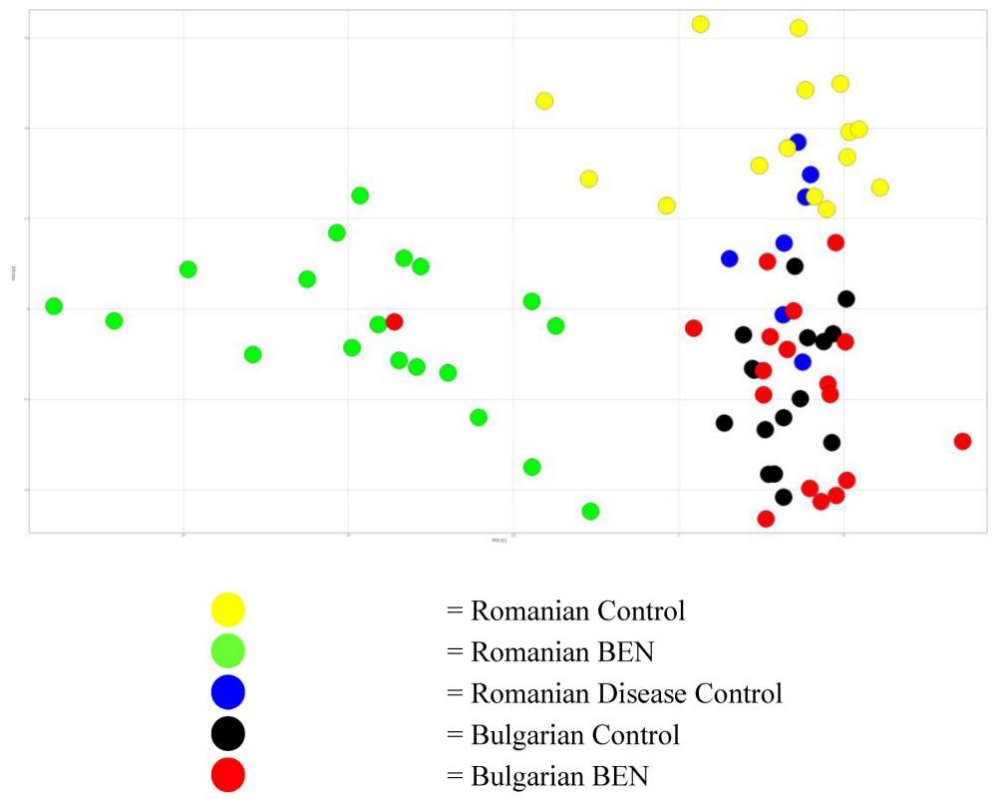
Scores plot of the first two components showing the separation of the samples from the Romanian BEN subjects compared to all other groups.

**Figure 4:**
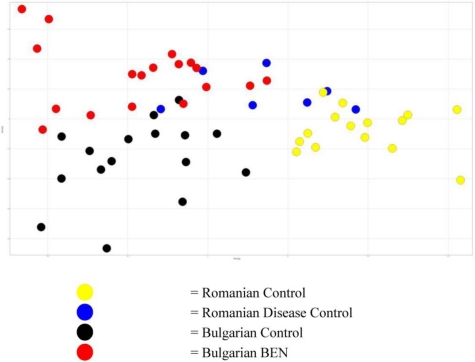
Scores plot of the second and third components with the Romanian BEN samples removed from the visualisation showing the clustering each of the other groups.

From the 3D scores plot in Figure 2 and the data shown in the 2D scores plots (Figure 3 and Figure 4), it was apparent that the variation in the data giving rise to the separation of the samples from the Romanian BEN subjects was not reflected in the samples from the other subjects. From examination of the NMR spectral data and comparison to available on-line metabolic profiling data sources the following observations were made. Many of the observations reflected those seen between the samples from Romanian BEN subjects and the Romanian control subjects. The urinary alterations included an increase relative to the samples from Bulgarian BEN subjects in acetate, lactate, glycine, myo-inositol, indolelactate (tentative), and glucose along with an increase to the region δ 0.90–1.00 typically attributed to amino acids (e.g., isoleucine), short chain organic acids and protein. A higher level of metabolites was observed in the samples from the Bulgarian BEN subjects compared to those from the Romanian BEN subjects and included citrate, TMAO and pyruvate. 

From assessment of these changes it appeared that the Romanian BEN subjects were displaying urinary changes that would be expected where damage to the tubular function had occurred, probably in the cortical region of the kidney. NMR data concerning organic acids is reminiscent of a Warburg effect. However, these same effects were not observed in the Bulgarian BEN subjects where the large TMAO alteration was observed. TMAO acts as a renal osmolyte and has been suggested to reflect alteration in the medulla. Whether these differences imply a different stage of the onset of BEN or a variation in the causative mechanism would require further study. Clustering of the Bulgarian BEN individual (Supplementary Table 2, case 15) in Romanian BEN scores space (Figure 2 and Figure 3) correlates with the NMR data showing organic aciduria, amino aciduria and glucosuria.

## 4. Discussion

Notably, urines of the three Romanian control subjects from hyperendemic villages and which clustered towards the well separated BEN group (Figure 2) had low creatinine concentration and the group included the person suspected as developing BEN and we recognise possible diagnostic potential.

Geographical differentiation in expression of BEN, revealed by measurement of urinary biomarkers and achieved through access to sophisticated analytical instrumentation, must raise the question of aetiology. The present pilot study implies that, if urinary biomarker expression of BEN differs between two discrete though adjacent localities in the Balkans, BEN could be a heterogeneous morbidity with respect to causal factors. For the present purpose, BEN refers just to the chronic bilateral renal atrophy which has been recognised for over half a century as hyperendemic in very specific parts of the Balkans. An assumption is that BEN has homogeneous aetiology across its particular Balkan foci, mainly one each in Romania and Bulgaria and several throughout the former Yugoslavia (currently in adjacent areas of Croatia and the Republika Srpska region of Bosnia, and in several southern parts of Serbia). Although the disease may have similar clinical manifestation as a progressive silent bilateral renal atrophy across the region, it may be prudent not to assume aetiological homogeneity. Rather frequently BEN is accompanied by transitional cell carcinoma of the upper urinary tract, different in type and location from renal cell carcinoma originating in the renal parenchyma. The reverse has not been observed. It would be unwise to assume, therefore, that the bilateral renal atrophy and urinary tract tumourigenesis are both caused by the same agent; tumourigenesis could be consequent on the progressive loss of renal function, occurring during the many years of silent development of BEN. Conventional urinalysis routinely used for chemical pathology diagnostic purposes has not revealed any biomarkers for the renal atrophy, but the present application of metabolomic methodology opens a way to view more sophisticated detail. Already our ^1^H NMR spectroscopic data offers new potential, and further detail may emerge as our HPLC/MS/MS data is studied. Hence, comparative analysis of the difference in composition of urine of Balkan healthy subjects in BEN villages and of those diagnosed with BEN by the most modern criteria [9,10], all carefully selected through international co-operation, could reveal a new level of diagnostic detail through application of the analytical power of ^1^H NMR spectroscopic and mass spectrometric methodologies to very small volumes of urine.

Historically, since BEN was first recognised in Bulgaria [5], several hypotheses were advanced from within the Balkans. A poisoning of the water by wartime munitions was thought plausible for regions of former conflict in Croatia and Serbia in the 2nd World War. Involvement of the arable weed birthwort (*Aristolochia clematitis*) was suggested in Serbia because of a somewhat analogous nephropathy in horses [11]. The biological activity of its mainly-seedborne toxin, aristolochic acid (AA), was even recognised on account of clinical trials as a human anti-tumour agent [12]. A viral infection has been proposed [13]. On account of isolation and structural characterisation of the nephrotoxic mycotoxin ochratoxin A (OTA) in South Africa in the 1960s [14], and its economic impact as the cause of a troublesome nephropathy affecting the Danish bacon industry during mid-20th century, this toxin was proposed as the cause also of BEN [15,16]. Concurrently, despite prevailing political barriers, UK study of nephrotoxic moulds at the MRC Toxicology Laboratory focused on food-spoilage fungi from BEN villages in Bulgaria and Romania. Fungi such as *Aspergillus ochraceus* and *Penicillium verrucosum* (formerly described as *P. viridicatum* [17]), expressing biosynthesis of OTA, were not apparent. However, Bulgarian isolates of a common food-spoilage mould (*Penicillium verrucosum var. cyclopium*) from the Bulgarian nephropathy region near Vratza were found to cause striking histopathological changes specifically in rat kidneys when cultured biomass or extract was administered *per os* [18]. The active ingredient(s) was not characterised and long-term exposure studies were not made, but potential necrotising and tumourigenic activities were diagnosed from the pyknotic and proliferative features. Since then, the topic has been studied further [19,20,21] but the toxin structure remains elusive. Extensive cytogenetic study of BEN has continued in Bulgaria [22], familial variations in lipid metabolism have been observed in well-documented hyperendemic populations in Southern Serbia [23], and interesting coincidence of disease patterns with superficial lignite coal deposits has raised questions about toxicity of leachate in drinking water [24].

In comprehensive metabonomic urinalysis from a 90-day OTA gavage study in rats, based on the protocol of the NTP study [25], potentially nephrocarcinogenic dose regimens (averaging 50 or 150 µg OTA/kg body weight/day during each week) provided for discrimination of treated animals from controls [26] according to ^1^H NMR spectroscopy findings. However, no discrimination was evident at the lowest dose (average 15 µg/kg/day), translating simply to ~1 mg/day for a human adult. Since such projected human exposure exceeds by about three orders of magnitude the exposure commonly measured in Balkan environments [27,28], the discriminators identified for higher OTA exposure in the rat in chronic [26,29] or acute [30] experiment would hardly be expected to have become apparent in BEN patients in the present study. Consequently we find no indicator of significant OTA exposure and are not persuaded concerning OTA as an aetiological factor in the bilateral renal atrophy of BEN. Similar conclusion has been made from other reasoning [31].

With the discovery of its potent nephrocarcinogenicity in male rats [25], OTA became a popular etiological candidate for BEN because it can readily be ingested via food spoilage, albeit usually in extremely small amounts, and similarities were seen by some researchers with mycotoxic porcine nephropathy [15,16]. Increasingly sensitive chromatographic methodology was also revealing OTA more widely as a food contaminant but this hardly fits the highly mosaic occurrence of BEN. OTA’s urinary tract carcinogenesis in rodents, targeting renal parenchyma, differs from the transitional cell carcinomas often associated with BEN, and the inconvenient disparity between the renal atrophy of BEN and the renal hypertrophy in porcine nephropathy is generally ignored by protagonists of ochratoxicosis A as a factor in BEN. However, DNA ploidy distribution in tumours associated with BEN matched the marked aneuploidy found in rat renal carcinomas caused by OTA [32], so a role in transitional cell tumourigenesis associated with BEN remains a possibility.

Aristolochic acid then became implicated as an aetiological factor in the so-called Chinese Herbs Nephropathy (CHN), recognised in Belgium among women taking a herbal–based slimming regimen in the 1990s [33]. Close similarities between CHN and BEN with regard to pathology were described by [34] and have focused new attention to a wider concept of AA nephropathy embracing both renal fibrosis and urinary tract tumours. A long-term experiment with rabbits given AA (0.1 mg/kg i.p) five times each week for 17 or 21 months caused anorexia, marked reduction in weight gain, doubling of kidney weight and changes in renal biological data [35]. After the longer period there was notable fibrosis in kidneys and two tumours were found, one in kidney and another in a ureter. In rats, a large AA dose (50 mg/kg/day for three days) elicited a toxic response [36], but renal function recovered within a month. Three renal carcinomas were found after six months, but there were none of the transitional cell carcinoma associated with BEN or characteristic of the Belgian CHN. 

Interesting new molecular findings have recently been published [37,38] concerning defined AA/DNA adducts isolated from urinary tract tumours of four Croatian BEN cases. DNA adduct structures matched those associated with TCC tumours of the Belgian CHN. The authors attributed both BEN and its associated urinary tract tumours to AA poisoning and this has become a persuasive proposition accepted somewhat uncritically [39,40,41]. Unfortunately, the geographic provenance of the analysed tissues was not given, so that it is not possible to estimate the incidence of *A. clematitis* in the environment of the BEN patients, relative to those without BEN, which could have influenced exposure to AA. Notably, chromatographic illustration of adducts showed one tumour to have had much greater incidence than the other three, and there is no indication as to whether adducts were in tumour parenchyma or were at least partly in nucleated cells of blood in the vascular compartment. To assist in confirming any possible causal specificity to the transitional cell carcinomas in some BEN patients, it would be good to have seen molecular genetic evidence from tumours in organs other than urological ones (e.g., lung) in BEN patients; smoking has been as prevalent in Balkan countries as in many other parts of the world. Also, the proposal that AA is an aetiological factor needs to be correlated with compelling evidence on natural exposure of individual patients to a carcinogenic dose. A poor fit has already been observed [42]. Also a differential exposure pattern must be explained between endemic and non-endemic settings, considering the geographically-unrestricted distribution of the weed plant. In strongly promoting AA as the cause of BEN [37], a clear differentiation from OTA was made according to genotoxic *versus* non-genotoxic modes of carcinogenicity, respectively, by selective use of literature. However, EFSA took a more ambivalent attitude to the current scientific literature on formation of covalent DNA adducts [43] and such a fundamental distinction can not now be sustained [44]. 

A survey was conducted by Croatian medical students using a questionnaire for elderly BEN patients with end-stage renal disease supported by haemodialysis at Slavonski Brod, and also to a group of apparently healthy residents in the historically-hyperendemic BEN village of Kaniza [45]. Whereas agricultural practice and socio-economics differed considerably from 20–30 years previously, people still recollected *Aristolochia* as a weed in wheatfields. Interestingly, the BEN patients recollected more frequent agricultural contamination by *A. clematitis* than did healthy residents in Kaniza. More details of data collection in the survey would have been helpful in eliminating potential bias in questioning patients with a mysterious terminal disease requiring haemodialysis for life-support, and to acknowledge the challenge of exploring long-retrospective memory. The study would have benefited from more statistical power. It was estimated that potentially-toxic historic AA-contamination of local wheat flour by *A. clematitis* seed could have occurred in Croatian endemic villages sufficient to match, through bread consumption, the daily 1 mg of AA associated with AA nephropathy cases in China. Unfortunately the cited reference for the latter could not be found. Also unfortunate is the mis-calculation [45] which underestimated the extent of Croatian bread contamination by *A. clematitis* seeds necessary to match AA intake in Belgian or Chinese human nephropathy by a factor of 7.33.

**Figure 5:**
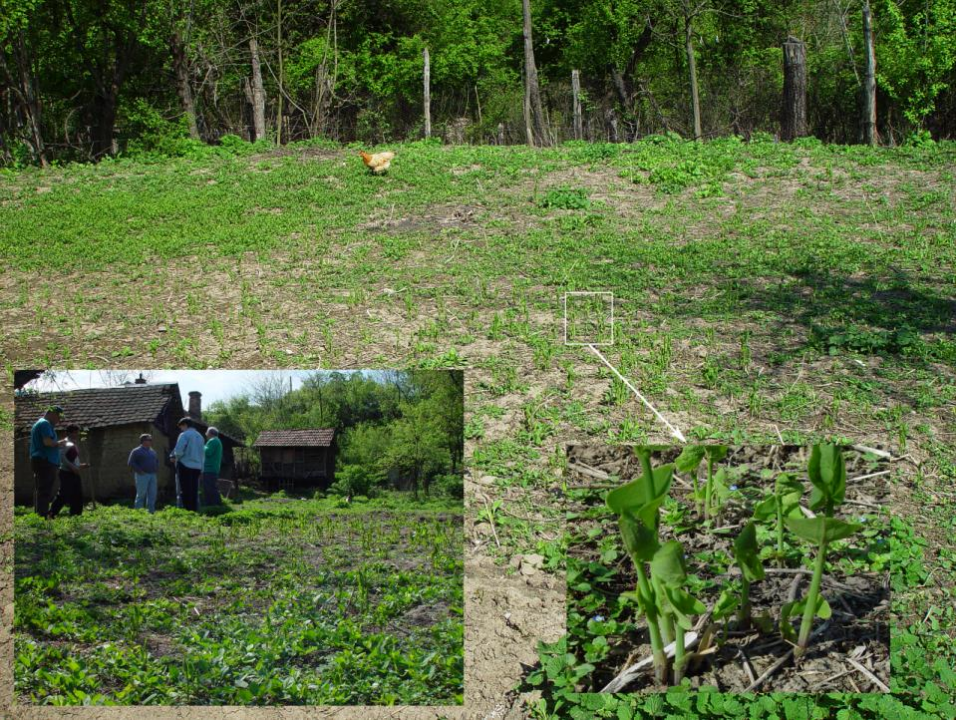
Scene at rear of house of former BEN patient in Erghevita, Romania showing re-growth of the dominant weed *Aristolochia clematitis*. May 2004.

Consequently, convincing evidence for natural toxigenic intake of AA in the specific nephropathy households of Croatian nephropathy villages, such as Kaniza at flood plain level near the Sava river, remains to be demonstrated. For this to apply also to the highly mosaic distribution of nephropathy households in hyperendemic villages across the several endemic areas in Balkan countries will require evidence which accommodates their heterogeneous topography and different agriculture. For example, in our experience, the historically hyperendemic Romanian village (Erghevita [46] has a much more undulating topography than that of Kaniza, and *A. clematitis* is a common weed there. Notably, Figure 5 illustrates in 2004 the extensive infestation in the garden of a household of a recently deceased male BEN patient. At first sight, although a striking coincidence, a plausible direct connection between plant and disease is difficult to envisage. The springtime infestation illustrated could hardly have persisted to full maturity so close to the house. Dried and powdered leaves are used by some people in rural communities, mixed with pig fat, for topical application for arthritic pain or bruises; leaf decoction (usually one or two leaves boiled in one litre of water) is also used by women for vaginal douching. However, such uses would imply very little or insignificant exposure to AA and be hardly focal to specific villages. Topical absorption of traces of AA might occur, sufficient to accumulate for detection as DNA adducts in tumours where impaired capacity for DNA repair could allow their accumulation. However, separate extensive GC-MS urinalysis of many samples from Romanian nephropathy villages by one of us (C.T.) has failed to find any trace of free AA, although spasmodic seasonal use of *A. clematitis* herbal preparations might in part be responsible for the negative results. In the situation illustrated in Figure 5, other ways of ingesting traces of AA might be via eggs or meat of chickens having seasonal access to seeding plants, but such routes and skin absorption remain to be substantiated. Claims of easy natural contamination of harvested wheat by seeds of *A. clematitis* are not assisted by illustration of the very green weed in a field from which the wheat had apparently long since been harvested [33]. The flour-contamination hypothesis is also contradicted by the hilly topography of the endemic, as well as non-endemic, villages in Romania, where wheat cultivation has been very limited. Other crop plants, like staple corn (*Zea mais*), are much more frequently cultivated there, but to what extent AA can contaminate the grain and enter the human food chain are open questions. Further, medicinal use of *A. clematitis* has consistently been reported only for Romania, although without any significant difference between endemic and non-endemic households. 

A study of use of herbal therapeutic remedies in Romania, focusing at the turn of the millennium on the same BEN endemic area as in the present study has concluded that there was no basis for currently invoking such practices as contributing a significant human exposure to AA [41]. A slightly higher usage by BEN patients was noted but there was no evidence that that rate occurred also before those people developed the morbidity. The occurrence of *A. clematitis* as a weed around some croplands was recognised (and of course we have seen that), but there was no evidence that harvested wheat was contaminated by the seeds, nor that grain cleaning at mills would have failed to remove such contaminants. AA was not found in any human plasma sample, although the limit of detection of the HPLC methodology was not stated. However, authors were influenced by the proposal [45] that in a Croatian context wheat flour would historically have been contaminated by *A. clematitis* seeds and that this could provide a significant intake of AA within a major dietary component. Unfortunately, although authors were informed of this error in 2007 it does not yet seem to have been corrected in the literature. Thus, in [41], authors were inadvertently misled in the extent to which citation of [45] could reasonably support their admission that ingestion of a nephrotoxic dose of AA from bread might have occurred in Romania in the 20th century. 

Currently, diametrically-opposed published convictions about OTA *versus* AA as the cause of BEN and associated TCC remain both philosophically unsatisfactory and unreconciled [37,38,47,48]. Sensational journalism [39] and uncritical attention to literature hardly fosters calm and rational consideration of all the complex factors in a disease that remains unexplained. It is important to satisfy the necessary evidence base in epidemiology [49].

The present study has been by collaborators with extensive experience with BEN in the field, and has been made conscious of various causative hypotheses and with minds completely open to new ideas, critical appraisal of current theories, and observing and listening to people in villages and clinics. Subjects enduring terminal morbidity deserve a fresh co-operative international approach to BEN. When the aetiology of BEN and its tumours is satisfactorily explained the facts about causal agent(s) and their quantitative exposure will be seen to fit comfortably with the rigorous metaphysical requirements of the classic Koch’s Postulates in defining a disease determinant. An example of this, concerning a natural toxicant and a mysterious morbidity, was the case of proving step by step that trace amounts of the lolitrem B metabolite of a cryptic obligate fungal endophyte in grass is the cause of “ryegrass staggers” in agricultural ruminants [50]. 

Design of this pilot study in 2003/4 was necessarily based on convenience sampling for logistic, ethical and economic reasons. However, this did not constrain the intended novel focus, namely to apply state-of-art metabolomic techniques to seek biomarkers of disease, but differentiation of BEN patients between Romanian and Bulgarian foci was not expected. The findings need confirmation, and people who suffer from a disease that has remained idiopathic for more than half a century deserve better understanding of the cause(s). Design now of a comprehensive study with the best international co-operation supported by Academies of Science and with access to appropriate methodology would seem to be justified.

## Conflict of Interest

Authors declare no conflict of interest.

## Supplementary Files

Supplementary File 1: PDF-Document (PDF, 160 KB)
