# Supplementary material for: Comparative 1H NMR Metabolomic Urinalysis of People Diagnosed with Balkan Endemic Nephropathy, and Healthy Subjects, in Romania and Bulgaria: A Pilot Study
Source: Toxins (Basel). 2011 Jul 4;3(7):815–33. doi: 10.3390/toxins3070815 (PMC3202861; doi:10.3390/toxins3070815)
Supplement: Supplementary File 1: — PDF-Document (PDF, 160 KB) [file toxins-03-00815-s001.pdf]

Supplementary Data

Table 1. Bulgaria controls. Urinalysis data. Units: creatinine (mmol/L), and other parameters (mmol/mmol creatinine).

Case	Creatinine	Ca	Urate	Prot	Na	K	PO_4	Urea
1	10.51	0.03	0.13	0.01	15.5	1.5	1.0	13.3
2	9.72	0.11	0.23	0.01	26.6	5.7	1.9	34.1
3	42.52	0.01	0.10	0.01	4.8	2.3	0.6	6.1
4	16.33	0.17	0.21	0.01	16.2	2.4	3.8	19.5
5	6.52	0.08	0.12	0.01	15.0	2.1	0.2	25.3
6	15.52	0.33	0.35	0.01	18.9	5.1	1.0	26.0
7	13.31	0.07	0.29	0.14	9.8	2.6	1.7	28.0
8	7.78	0.12	0.31	0.02	31.4	14.3	0.7	18.2
9	8.80	0.08	0.22	0.13	24.8	11.2	0.7	15.3
10	13.82	0.18	0.29	0.01	14.5	9.5	1.4	30.3
11	11.58	0.12	0.19	0.01	16.0	3.2	1.2	25.1
12	8.13	0.19	0.25	0.01	19.8	6.5	1.5	31.0
13	7.83	0.11	0.45	0.01	33.7	8.2	1.9	36.2
14	14.85	0.17	0.26	0.28	16.6	4.7	1.6	20.5
15	19.58	0.22	0.23	0.01	12.8	1.9	0.9	14.0
16	15.86	0.06	0.23	0.01	19.6	2.9	2.1	29.8
17	14.45	0.10	0.24	0.17	13.4	4.1	1.5	15.2
18	13.14	0.15	0.20	0.04	13.1	2.1	1.0	10.4
19	15.23	0.09	0.41	0.01	13.8	3.3	1.3	29.3
20	4.4	0.46	0.34	0.03	39.1	7.3	2.6	36.8
21	5.60	0.17	0.25	0.02	22.3	2.3	1.1	27.2

Table 2. Bulgaria BEN. Urinalysis data. Units: creatinine (mmol/L), and other parameters (mmol/mmol creatinine).

Case	Creatinine	Calcium	Urate	Protein	Sodium	Potassium	PO_4	Urea
1	9.46	0.23	0.12	0.02	20.43	5.4	2.53	37.35
2	17.31	0.02	0.01	0.02	7.77	3.6	1.23	31.65
3	5.46	0.15	0.22	0.03	47.18	4.47	2.69	43.52
4	8	0.35	0.13	0.01	23.62	7.36	2.08	45.85
5	5.62	0.32	0.18	0.03	42.34	3.56	3.95	52.03
6	6.36	0.15	0.12	0.09	35.27	7.51	2.79	46.44
7	7.67	0.08	0.13	0.07	27.25	10.84	7.43	51.74
8	11.85	0.41	0.89	0.41	14.25	4.88	1.17	34.97
9	3.79	0.66	0.23	0.09	63.57	7.62	2.78	62.4
10	4.43	0.25	0.25	0.02	50.7	3.77	2.28	36.9
11	9.1	0.08	0.11	0.02	20.4	8.18	1.95	28.82
12	10.07	0.23	0.25	0.01	26.93	7.51	1.82	29.47
13	4.37	0.38	0.13	0.03	51.07	6.86	3.17	50.56
14	3.41	0.22	0.13	0.02	29.99	7.17	3.96	51.83
15	3.79	0.07	0.29	0.23	27.57	5.87	2.21	36.67
16	6.46	0.02	0.22	0.07	25.46	5.68	1.29	35.1
17	5.6	0.6	0.19	0.01	37.71	8.4	3.34	46.49
18	20.66	0.24	0.04	0.01	14.2	3.28	1.97	24.15
19	23.24	0.02	0.16	0.01	5.16	6.74	1.26	19.7

Table 3. Romania controls. Urinalysis data. Units: creatinine (mmol/L), and other parameters (mmol/mmol creatinine).

**Case**	**Age**	**Sex**	**Creatinine**	**Ca**	**Urate**	**Protein**	**Na**	**K**	**PO_4**	**Urea**
D-T Severin region										
1	38	Female	10.04	0.37	0.36	0.008	32.47	3.79	5.05	30.4
2	36	Male	11.07	0.98	0.05	0.006	20.09	2.32	2.11	44.64
3	58	Female	4.11	0.29	0.56	0.88	13.59	5.86	1.98	34.38
4	60	Male	7.19	0.08	0.61	0.01	16.65	4.89	3.68	26.12
5	37	Male	13.01	0.25	0.35	0.01	19.63	4.08	1.37	26.25
Erghevita										
6	62	Male	7.5	0.02	0.16	0.14	9.31	3.01	3.67	17.76
7	6	Male	5.81	0.05	0.14	0.02	32.93	11.23	4.05	68.96
8	36	Female	7.32	0.13	0.18	0.07	17.01	3.91	1.46	29.5
9	49	Male	6.12	0.11	0.25	0.01	24.21	6.01	1.71	30.22
10	31	Male	2.96	0.13	0.33	0.02	37.28	2.14	2.63	38.01
11	49	Male	6.75	0.6	0.31	0.01	38.12	9.65	1.99	34.91
12	66	Male	1.39	0.19	0.31	0.09	34.94	6.99	6.75	32.61
13	72	Male	7.77	0.2	0.07	0.01	19.22	3.2	1.92	20.75
14	60	Female	0.71	0.26	0.31	0.09	61.63	8.78	15.44	41.6
15	63	Male	13.02	0.08	0.29	0.02	16.42	8.33	1.69	8.17
16	64	Male	0.94	0.13	0.35	0.07	42.62	5.76	16.93	27.64
17	27	Female	6.85	0.15	0.32	0.01	29.12	6.47	2.23	26.05
18	46	Female	3.26	0.59	0.34	0.02	40.47	8.09	3.07	38.44
Bistrita										
19#	12	Female	12.12	0.21	0.14	0.01	17.66	2.39	3.24	50.27
20+	40	Male	11.15	0.03	0.01	0.02	25.02	3.77	1.69	26.1
Rogova										
21	60	Female	4.50	0.08	0.13	0.04	34.19	2.89	1.57	42.18
22*	7	Male	8.73	0.34	0.17	0.01	11.7	2.64	2.08	61.3
23*	4	Male	7.17	0.54	0.39	0.01	20.09	4.05	1.79	60.13
24*	25	Female	15.66	1.11	0.08	0.01	12.26	2.55	1.4	34.54
25*	35	Male	14.16	0.26	0.06	0.01	10.45	2.68	1.34	28.46

# and + Daughter and brother, respectively, of BEN patient

* Resident in same household as a female BEN patient (haemodialysis, age 54)

Table 4. Romanian subjects receiving haemodialysis, or peritoneal dialysis, in Drobeta Turnu Severin for BEN or other nephropathy; urinalysis data and some personal details. Units: creatinine (mmol/L), and other parameters (mmol/mmol creatinine).

Case	Age	Sex	Diagnosis	Months on dialysis	Residence	In- or out-patient	Creatinine	Ca	Urate	Prot	Na	K	PO_4	Urea
2003														
1	60	M	BEN	84	Patulele	Out	7.13	0.1	0.03	0.11	15.9	1.71	0.47	14.19
2	62	F	BEN	20	Rogova	Out	5.87	0.05	0.16	0.36	16.81	9.08	1.87	14.99
3	65	F	BEN	25	Devesel-Mileni	Out	2.84	0.16	0.2	0.21	44.87	5.16	1.29	26.21
5	69	M	BEN	15	Corcova-Croica	Out	5.01	0.12	0.2	0.3	51.66	2.87	1.38	24.26
6	64	F	BEN	2	Prunisor	Out	2.01	0.37	0.4	0.26	44.17	6.8	1.26	27.18
7	77	M	BEN	60	Rogova-Poroinita	Out	6.84	0.03	0.16	0.5	20.07	2.14	0.95	13.49
8	65	F	BEN	22	Rogova-Poroinita	Out	12.81	0.02	0.04	0.13	5.56	1.2	0.45	7.27
9	75	M	BEN	17	Fintina Domneaasca	Out	5.99	0.08	0.17	0.25	26.6	2.36	1.51	22.11
10	62	F	BEN	2	Strehaia-Stancesti	Out	2.91	0.11	0.26	0.43	25.45	3.69	0.93	18.44
11	55	F	Polycystic kidney	12	Dr. Tr. Severin	Out	2.3	0.1	0.15	0.30	47.3	7.31	1.11	31.7
12	45	F	BEN	1	Rogova-Poroinita	In	2.27	0.15	0.29	0.31	46.95	3.91	0.94	26.9
13	46	M	Chronic glomerulo-nephritis	14	Dr.Tr. Severin	Out	5.76	0.04	0.08	0.64	17.92	2.44	0.71	11.4
14	52	M	BEN	62	Bistrita	In	1.66	0.41	0.2	0.5	83.77	3.35	1.4	34.18
15	52	M	BEN	14	Dr. Tr. Severin	In	6.90	0.03	0.17	0.18	11.17	4.56	1.73	21.24
16	33	F	BEN	4	Bistrita	Out	3.52	0.15	0.3	0.32	26.76	3.34	1.47	36.79
17	58	F	BEN	82	Valea Izvorului de Sus	Out	2.15	0.27	0.26	0.64	69.41	5.1	1.64	16.84
18	70	M	BEN	38	Livezile	In	6.16	0.01	0.1	0.53	13.99	2.8	0.59	17.14
19	48	M	Chronic pyelo-nephritis	20	Manu	In	9.99	0.02	0.11	0.26	4.67	3.45	1.41	24.03
20	34	F	BEN	1	Rogova-Poroinita	In	5.59	0.03	0.06	0.56	12.41	1.16	1.16	12.41
21	66	F	BEN	71	Bistrita	In	3.74	0.14	0.17	0.33	26.08	3.01	3.01	25.41
23	65	F	BEN	?	?	Out	1.58	0.3	0.21	0.71	84.99	4.18	0.97	16.72
24	54	F	BEN	?	?	Out	5.32	0.08	0.21	0.42	23.04	5.03	1.38	18.27
25	67	M	BEN	?	?	Out	3.62	0.18	0.23	0.12	36.54	2.61	0.87	22.51
26	65	M	BEN	?	?	Out	3.58	0.06	0.22	0.47	31.84	4.1	1.05	19.54
2004														
27 *	71	F	BEN	49	Livezile	Out	2.31	0.09	0.24	0.27	41.91	2.35	4.47	19.31
28	66	M	BEN	64	Simian	Out	3.27	0.1	0.27	1.26	33.45	6.08	4.93	20.95
29	65	F	BEN	2	Rogova	Out	6.02	0.07	0.17	0.22	16.35	3.67	3.01	30.86
30 *	46	F	BEN	12	Rogova-Poroinita	Out	1.9	0.3	0.17	0.46	55.48	5.37	6.3	21.96

* same as cases 18 and 12, respectively, 2003

Table 5. Romanian control subjects with urinary tract tumours, but not with BEN, living in or near Timisoara (distant from the BEN area): urinalysis data and some personal details. Units: creatinine mmol/L; other components, µmol/mmol creatinine.

	Age	Sex	Residence	Tumour	Creatinine	Calcium	Urate	Protein	Sodium	Potassium	Phosphate	Urea
1	70	Male	Deva-Hunedoara	Left urinary tract tumour	12.62	0.22	0.14	0.19	18.44	2.5	3.99	54.11
2	66	Male	Timisoara	Prostate adenoma	11.67	0.11	0.16	0.03	26.2	3.57	2.0	27.32
3	58	Female	Hartagani-Hunedoara	Bladder and right kidney tumours	5.08	0.03	0.18	0.04	33.78	2.63	1.63	42.33
4	75	Male	Timisoara	Peri-ureteral adenoma	9.08	0.35	0.25	0.01	12.01	2.5	2.87	66.96
5	75	Male	Lovrin-Timisoara	Prostate tumour	8.21	0.53	0.43	0.01	20.44	4.03	2.32	63.18
6	60	Male	Timisoara	Prostate adenoma	17.32	1.12	0.22	0.007	12.71	2.54	1.71	37.41
7	69	Male	Jimbolia-Timisoara	Prostate adenoma and right kidney tumour	15.05	0.26	0.21	0.005	10.8	2.79	1.54	31.07
